# Blood eosinophils as a biomarker of future COPD exacerbation risk: pooled data from 11 clinical trials

**DOI:** 10.1186/s12931-020-01482-1

**Published:** 2020-09-17

**Authors:** Dave Singh, Jadwiga A. Wedzicha, Salman Siddiqui, Alberto de la Hoz, Wenqiong Xue, Helgo Magnussen, Marc Miravitlles, James D. Chalmers, Peter M. A. Calverley

**Affiliations:** 1 Medicines Evaluation Unit, University of Manchester, Manchester University NHS Foundation Trust, Manchester, UK; 2 Respiratory Division, National Heart and Lung Institute, Imperial College London, London, UK; 3 NIHR Leicester Biomedical Research Centre: Respiratory Theme, and Department of Respiratory Sciences, University of Leicester, Leicester, UK; 4 Boehringer Ingelheim International GmbH, Ingelheim am Rhein, Germany; 5 Boehringer Ingelheim Pharmaceuticals, Inc., Ridgefield, CT USA; 6 Pulmonary Research Institute at Lung Clinic Grosshansdorf, Grosshansdorf, Germany; 7 Pneumology Department, Hospital Universitari Vall d’Hebron/Vall d’Hebron Research Institute (VHIR), CIBER de Enfermedades Respiratorias (CIBERES), Barcelona, Spain; 8 Scottish Centre for Respiratory Research, University of Dundee, Ninewells Hospital and Medical School, Dundee, UK; 9 Clinical Science Centre, Institute of Ageing and Chronic Disease, University of Liverpool, Liverpool, UK

**Keywords:** Eosinophils, Exacerbations, ICS, Rate ratio, Clinically irrelevant, Randomized controlled trials, Pooled

## Abstract

**Background:**

Chronic obstructive pulmonary disease (COPD) is characterised by progressive airflow limitation and chronic inflammation. Predicting exacerbations of COPD, which contribute to disease progression, is important to guide preventative treatment and improve outcomes. Blood eosinophils are a biomarker for patient responsiveness to inhaled corticosteroids (ICS); however, their effectiveness as a predictive biomarker for COPD exacerbations is unclear.

**Methods:**

This post hoc analysis pooled data from 11 Boehringer Ingelheim-sponsored Phase III and IV randomised COPD studies with similar methodologies. Exacerbation data were collected from these studies, excluding patients from the ICS withdrawal arm of the WISDOM® study. Patients were grouped according to their baseline blood eosinophil count, baseline ICS use and number of exacerbations in the year prior to each study.

**Results:**

Exacerbation rate data and baseline eosinophil count were available for 22,125 patients; 45.6% presented with a baseline blood eosinophil count of ≤ 150 cells/μL, 34.3% with 150–300 cells/μL and 20.1% with > 300 cells/μL. The lowest exacerbation rates were observed in patients with ≤ 150 cells/μL, with small increases in exacerbation rate observed with increasing eosinophil count. When stratified by exacerbation history, the annual rate of exacerbations for patients with 0 exacerbations in the previous year increased in line with increasing eosinophil counts (0.38 for ≤ 150 cells/μL, 0.39 for 150–300 cells/μL and 0.44 for > 300 cells/μL respectively). A similar trend was identified for patients with one exacerbation in the previous year, 0.62, 0.66 and 0.67 respectively. For patients with ≥ 2 exacerbations, exacerbation rates fluctuated between 1.02 (≤ 150 cells/μL) to 1.10 (150–300 cells/μL) and 1.07 (> 300 cells/μL). Higher exacerbation rates were noted in patients treated with ICS at baseline (range 0.75 to 0.82 with increasing eosinophil count) compared with patients not on ICS (range 0.45 to 0.49).

**Conclusion:**

We found no clinically important relationship between baseline blood eosinophil count and exacerbation rate. Hence, the current analysis does not support the use of blood eosinophils to predict exacerbation risk; however, previous exacerbation history was found to be a more reliable predictor of future exacerbations.

**Trial registration:**

ClinicalTrials.gov Identifiers: NCT00168844, NCT00168831, NCT00387088, NCT00782210, NCT00782509, NCT00793624, NCT00796653, NCT01431274, NCT01431287, NCT02296138 and NCT00975195.

**Graphical abstract:**

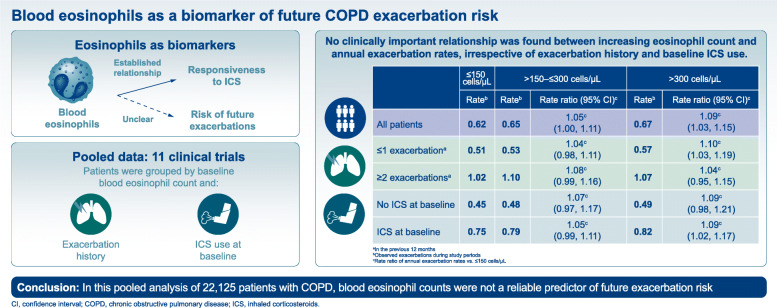

## Plain language summary

People with chronic obstructive pulmonary disease often start treatment with either a long-acting muscarinic antagonist or a long-acting β_2_-agonist. Doctors can prescribe these treatments together (long-acting muscarinic antagonist/long-acting β_2_-agonist) if needed. Other medicines called inhaled corticosteroids can be added as required. Measuring the number of eosinophils, a type of white blood cell, in the blood can help to predict which people will benefit most from inhaled corticosteroid treatment.

People with chronic obstructive pulmonary disease sometimes experience a worsening of their symptoms, known as an exacerbation. Eosinophil levels may be useful to predict the risk of exacerbations. We studied the results from 11 clinical trials, involving 22,125 people with chronic obstructive pulmonary disease. We looked at how many exacerbations people had during these trials and whether this was linked to the level of eosinophils in their blood, their previous history of exacerbations, or whether they had been treated with inhaled corticosteroids before.

Overall, we found that a previous history of exacerbations predicted the future rate of exacerbations. We did not find a clear link between the rate of exacerbations and eosinophil levels.

## Background

Characterised by progressive airflow limitation and chronic inflammation, chronic obstructive pulmonary disease (COPD) is a major source of morbidity and mortality that mainly affects individuals above the age of 35 years [1, 2]. COPD exacerbations, defined as acute worsening of respiratory symptoms requiring a change in treatment [3], are important events that contribute to disease progression. In particular, severe exacerbations (defined as events requiring hospitalisation) are associated with a significantly worse survival and reduced quality of life [3]. The Global Initiative for Chronic Obstructive Lung Disease (GOLD) strategy report recommends that inhaled corticosteroids (ICS), used in combination with long-acting bronchodilator(s), are a useful treatment for patients with a history of exacerbations [4, 5]. GOLD also supports the use of blood eosinophil counts as a biomarker in clinical practice to predict the likelihood of ICS benefit in terms of preventing future exacerbations [4].

Predicting exacerbations is important for clinicians treating patients with COPD and for designing clinical trials. At present, the strongest predictor of future COPD exacerbations is the past history of moderate and severe exacerbations [4, 6]. Exacerbation risk is also associated with an increase in symptom severity and worse lung function [7]. Additionally, characteristics such as female sex, older age, lower forced expiratory volume in 1 s (FEV_1_) and persistent cough have been shown to be predictive of higher exacerbation rates [7, 8]. However, while a number of biomarkers have been studied to predict exacerbations in COPD [3, 9–11], none are currently used in clinical practice.

Randomised controlled trials (RCTs) in COPD patients have shown that blood eosinophil counts predict the response to ICS [12, 13]. Consequently, blood eosinophil counts have utility in clinical practice to direct ICS use in COPD patients, and as a biomarker in clinical trials to identify subgroups with different responses to anti-inflammatory drugs [14]. However, publications investigating the relationship between blood eosinophils and exacerbation risk have produced conflicting results [15–19]. Some observational cohort studies have reported that higher eosinophil levels are associated with future COPD exacerbations [15–17], but other studies suggest that there is no relationship [18, 19].

RCTs of ICS-containing combination treatments in patients with a history of exacerbations have consistently reported that there is no increase in exacerbation rates at higher blood eosinophil counts in patients allocated to receive ICS treatment, while increased exacerbation rates are observed at higher blood eosinophil counts in patients allocated to receive bronchodilator treatment without ICS [20–22]. This suggests that the use of ICS obscures any relationship between blood eosinophil counts and exacerbation rates.

Another key factor that affects the relationship between eosinophil levels and future exacerbations is the degree of study population enrichment with patients at increased risk of future exacerbations. Previous RCTs of ICS-containing combination treatments demonstrating a relationship between blood eosinophil counts and exacerbation rates (in patients randomised to non-ICS treatment) have included patients with a history of exacerbations in the previous year [20]. Furthermore, analysis of the ECLIPSE and COPDgene studies showed that the relationship between exacerbation risk and eosinophil count was clearest in patients with ≥ 2 exacerbations in the last year [17].

The aim of the present analysis was to further investigate whether blood eosinophil counts predict future rate of exacerbations by pooling data from a large number of clinical trials. These included patients with COPD who experienced exacerbations at different frequencies and of varying severity. Patients were stratified by recognised eosinophil cut-off levels at baseline [23, 24] and the rate of exacerbations was compared between eosinophil subgroups. Exacerbation rates for different eosinophil subgroups were assessed according to exacerbation history and ICS use at baseline.

## Methods

### Study design

Data from 11 previously published clinical studies were pooled in the current analysis [25–32]. These studies were a collection of Phase III and IV multicentre, double-blindRCTs where patients were treated with tiotropium, olodaterol or tiotropium/olodaterol combination therapy (ClinicalTrials.gov: NCT00168844, NCT00168831, NCT00387088, NCT00782210, NCT00782509, NCT00793624, NCT00796653, NCT01431274, NCT01431287, NCT02296138), as well as the WISDOM® ICS withdrawal study (NCT00975195). The inclusion and exclusion criteria have been described in detail previously [25–32]. Briefly, patients were included if they were aged ≥ 40 years with a diagnosis of COPD, had a smoking history of > 10 pack-years, a post-bronchodilator FEV_1_ of < 80% predicted (or < 60%) [26] and a post-bronchodilator FEV_1_/forced vital capacity of < 70%. All studies excluded patients with a confirmed diagnosis of asthma. All studies were conducted in accordance with the Declaration of Helsinki and Good Clinical Practice guidelines.

The current analysis focuses on patients with COPD who had documented eosinophil counts at baseline. Studies were included if they recorded exacerbations through the entire study period. The withdrawal arm of the WISDOM trial [31] was excluded from the analysis to avoid including patients with mandated ICS withdrawal. Patients on ICS at baseline continued ICS use during their respective trials.

### Statistical analysis

This post hoc analysis investigated the association between different blood eosinophil count categories (≤ 150 cells/μL, > 150–≤ 300 cells/μL and > 300 cells/μL) and the annual rate of moderate-to-severe exacerbations.

Data from patients across all studies were pooled and exacerbation rate data were analysed according to patients’ baseline eosinophil count (≤ 150 cells/μL, > 150–≤ 300 cells/μL or > 300 cells/μL), exacerbation history (stratified as either ‘infrequent exacerbators’ [≤ 1 moderate exacerbation and no severe exacerbations in the previous year] or ‘frequent exacerbators’ [≥ 2 moderate exacerbations or ≥ 1 severe exacerbation in the previous year]), and ICS use at baseline or according to both ICS use at baseline and exacerbation history. COPD exacerbations were counted from the start to the end of treatment. Patients were followed for the duration of each clinical trial (a minimum of 48 weeks).

Annual rates of exacerbations were assessed using negative binomial models with treatment exposure as offset, adjusted for treatment, study, ICS use at baseline, region, GOLD stage, smoking status, baseline eosinophil count, and number of exacerbations in previous year as covariates.

## Results

### Patient demographics

**Fig. 1:**
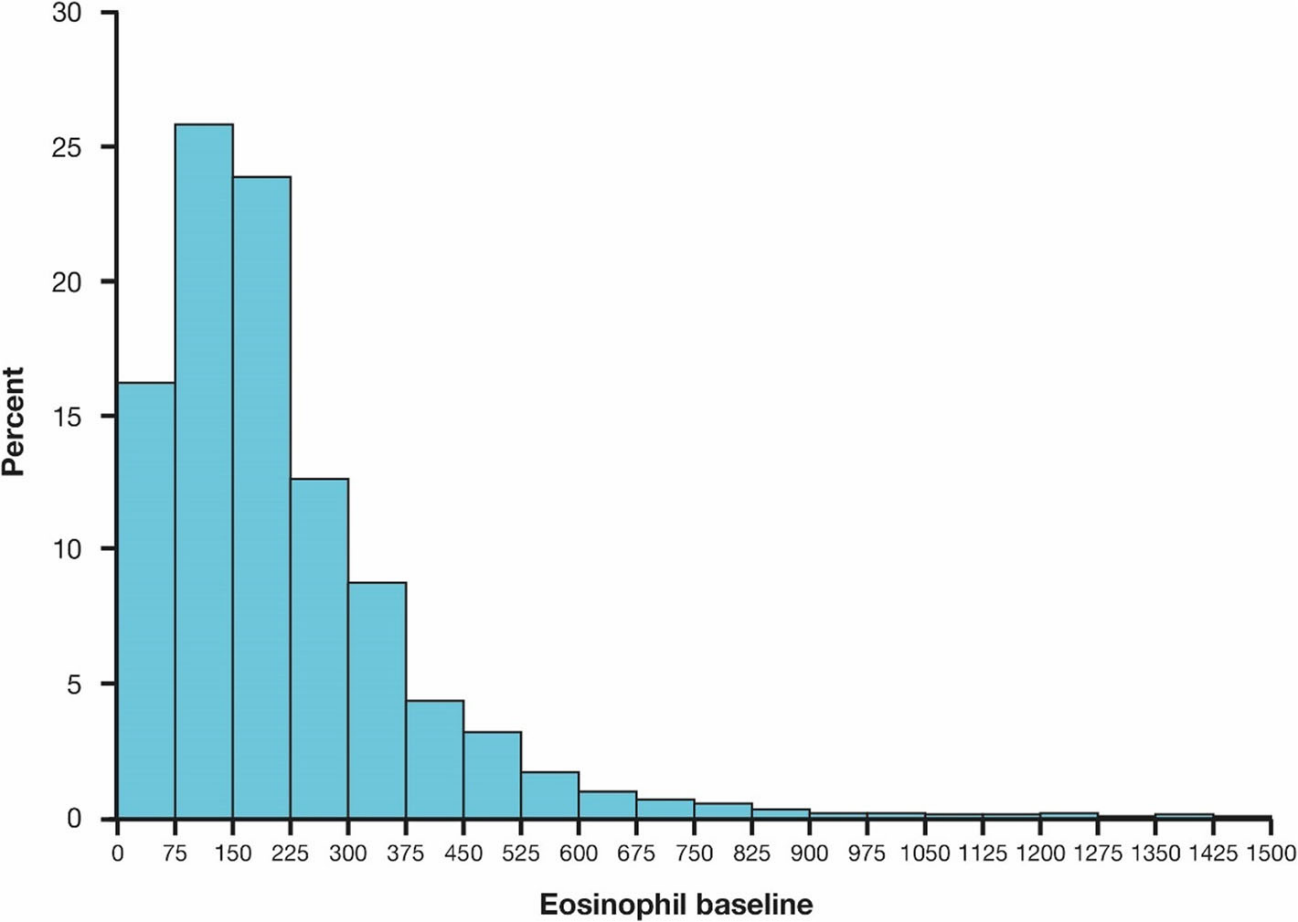
Baseline blood eosinophil levels in the total population. Baseline blood eosinophil count from 22,125 patients with accompanying exacerbation data

**Fig. 2:**
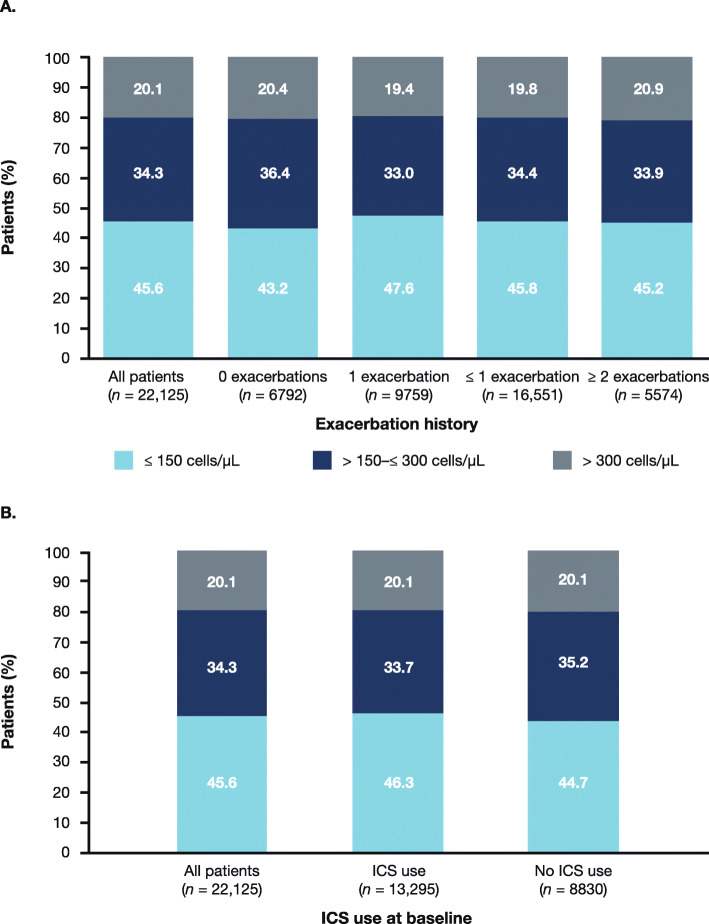
**a** Eosinophil count at baseline by exacerbation history. **b** Eosinophil count at baseline by ICS use. ICS, inhaled corticosteroids

From the pooled population, baseline eosinophil data were collected from 22,125 patients who also had exacerbation rate data (Fig. 1). The proportion of patients within each eosinophil subgroup was similar, irrespective of ICS use at baseline or exacerbation history (Fig. 2a and b). At baseline, the eosinophil count was ≤ 150 cells/μL in 10,096 patients (45.6%), > 150–≤ 300 cells/μL in 7581 patients (34.3%) and > 300 cells/μL in 4448 patients (20.1%). Median eosinophil count at baseline was 170.0 cells/μL.

Baseline characteristics are shown in Table 1. Mean age, gender split, smoking status, number of pack-years and incidence of moderate-to-severe exacerbations in the previous year were comparable between the three eosinophil subgroups. The majority of patients (74.1%) were male; 63.2% were ex-smokers and 36.8% were current smokers.

**Table 1:** Baseline patient characteristics

Characteristic	≤ 150 cells/μL (***n*** = 10,096)	> 150–≤ 300 cells/μL (***n*** = 7581)	> 300 cells/μL (***n*** = 4448)	Total (***N*** = 22,125)
Male, n (%)	7120 (70.5)	5694 (75.1)	3584 (80.6)	16,398 (74.1)
Mean age, years (SD)	65.3 (8.6)	65.0 (8.6)	64.6 (8.7)	65.1 (8.6)
Smoking status, n (%)				
Never smoked	0 (0.0)	0 (0.0)	1 (0.0)	1 (0.0)
Ex-smoker	6299 (62.4)	4788 (63.2)	2897 (65.1)	13,984 (63.2)
Current smoker	3797 (37.6)	2793 (36.8)	1550 (34.8)	8140 (36.8)
Smoking history, mean pack-years (SD)	45.6 (26.0)	45.7 (25.3)	45.0 (25.7)	45.5 (25.7)
Moderate-to-severe exacerbations in previous year, n (%)				
0	2850 (28.2)	2416 (31.9)	1347 (30.3)	6613 (29.9)
1	4672 (46.3)	3229 (42.6)	1907 (42.9)	9808 (44.3)
2	1565 (15.5)	1195 (15.8)	709 (15.9)	3469 (15.7)
3	533 (5.3)	385 (5.1)	251 (5.6)	1169 (5.3)
4	220 (2.2)	145 (1.9)	107 (2.4)	472 (2.1)
> 4	256 (2.5)	211 (2.8)	127 (2.9)	594 (2.7)
Severe exacerbations in previous year, n (%)				
0	8265 (81.9)	6362 (83.9)	3683 (82.8)	18,310 (82.8)
1	1501 (14.9)	970 (12.8)	614 (13.8)	3085 (13.9)
2	258 (2.6)	198 (2.6)	115 (2.6)	571 (2.6)
3	44 (0.4)	31 (0.4)	19 (0.4)	94 (0.4)
4	19 (0.2)	12 (0.2)	8 (0.2)	39 (0.2)
> 4	9 (0.1)	8 (0.1)	9 (0.2)	26 (0.1)
Mean pre-bronchodilator FEV_1_ at screening; L (SD)	1.15 (0.45)	1.18 (0.46)	1.16 (0.46)	1.16 (0.46)
ICS use at baseline, n (%)	6149 (60.9)	4477 (59.1)	2669 (60.0)	13,295 (60.1)

*FEV*_*1*_ Forced expiratory volume in 1 s; *ICS* Inhaled corticosteroids; *SD* Standard deviation.

### Exacerbation rate by eosinophil count and exacerbation history

In the total patient population, prospective annual exacerbation rates observed during the studies were numerically similar across the three eosinophil subgroups (≤ 150 cells/μL, 150–300 cells/μL and > 300 cells/μL; range: 0.62–0.67). When compared with patients with a baseline eosinophil count of ≤ 150 cells/μL, patients in the > 150–≤ 300 cells/μL and > 300 cells/μL subgroups had exacerbation rate ratios (RRs) of 1.05 (95% confidence interval [CI] 1.00, 1.11) and 1.09 (95% CI 1.03, 1.15), respectively (Table 2).

**Table 2:** Rate ratio of annual exacerbation rates according to exacerbation history by baseline eosinophil count (cells/μL)

		≤ 150 cells/μL	> 150–≤ 300 cells/μL	> 300 cells/μL
All	All patients *n* = 22,125	Rate: 0.62	Rate: 0.65 RR: 1.05 (95% CI 1.00, 1.11)	Rate: 0.67 RR: 1.09 (95% CI 1.03, 1.15)
Exacerbation history	0 exacerbations *n* = 6792	Rate: 0.38	Rate: 0.39 RR: 1.01 (95% CI 0.90, 1.12)	Rate: 0.44 RR: 1.14 (95% CI 1.00, 1.30)
	1 exacerbation *n* = 9759	Rate: 0.62	Rate: 0.66 RR: 1.07 (95% CI 0.99, 1.15)	Rate: 0.67 RR: 1.10 (95% CI 1.01, 1.19)
	≤ 1 exacerbation *n* = 16,551	Rate: 0.51	Rate: 0.53 RR: 1.04 (95% CI 0.98, 1.11)	Rate: 0.57 RR: 1.10 (95% CI 1.03, 1.19)
	≥ 2 exacerbations *n* = 5574	Rate: 1.02	Rate: 1.10 RR: 1.08 (95% CI 0.99, 1.16)	Rate: 1.07 RR: 1.04 (95% CI 0.95, 1.15)

RR versus ≤ 150 cells/μL eosinophil subgroup.

*CI* Confidence interval; *RR* Rate ratio.

In patients with ≤ 1 exacerbation in the previous year, annual exacerbation rates ranged from 0.51 to 0.57 (Table 2). When compared with patients with a baseline eosinophil count of ≤ 150 cells/μL, patients in the > 150–≤ 300 cells/μL and > 300 cells/μL subgroups had exacerbation RRs of 1.04 (95% CI 0.98, 1.11) and 1.10 (95% CI 1.03, 1.19), respectively. As shown in Table 2, similar patterns were identified in patients with 0 or 1 exacerbation in the previous year.

In patients with ≥ 2 exacerbations in the previous year, annual exacerbation rates ranged from 1.02 to 1.10 (Table 2). When compared with patients with a baseline eosinophil count of ≤ 150 cells/μL, patients in the > 150–≤ 300 cells/μL and > 300 cells/μL subgroups had RRs of 1.08 (95% CI 0.99, 1.16) and 1.04 (95% CI 0.95, 1.15), respectively.

### Exacerbation rate in patients stratified by ICS use at baseline

In the pooled analysis, 13,295 patients were receiving ICS at baseline; 8830 patients were not receiving ICS at baseline. Higher annual exacerbation rates were observed in patients receiving ICS at baseline (range 0.75 to 0.82 exacerbations) compared with non-ICS users (range 0.45 to 0.49) (Fig. 2b**,** Table 3).

**Table 3:** Rate ratio of annual exacerbations according to ICS use by baseline eosinophil count (cells/μL)

		≤ 150 cells/μL	> 150–≤ 300 cells/μL	> 300 cells/μL
Baseline ICS use	No *n* = 8830	Rate: 0.45	Rate: 0.48 RR: 1.07 (95% CI 0.97, 1.17)	Rate: 0.49 RR: 1.09 (95% CI 0.98, 1.21)
	Yes *n* = 13,295	Rate: 0.75	Rate: 0.79 RR: 1.05 (95% CI 0.99, 1.11)	Rate: 0.82 RR: 1.09 (95% CI 1.02, 1.17)

RR versus ≤ 150 cells/μL eosinophil subgroup.

*CI* Confidence interval; *ICS* Inhaled corticosteroids; *RR* Rate ratio.

For patients on ICS at baseline, when compared with the ≤  150 cells/μL group, patients in the > 150–≤ 300 cells/μL and > 300 cells/μL subgroups had exacerbation RRs of 1.05 (95% CI 0.99, 1.11) and 1.09 (95% CI 1.02, 1.17), respectively. For non-ICS patients, when compared with the ≤ 150 cells/μL group, patients in the > 150–≤ 300 cells/μL and > 300 cells/μL subgroups had exacerbation RRs of 1.07 (95% CI 0.97, 1.17) and 1.09 (95% CI 0.98, 1.21), respectively (Table 3).

### Exacerbation rate in patients stratified by exacerbation history before study start and ICS use at baseline

Table 4 shows the annual exacerbation rates stratified by exacerbation history and ICS use at baseline. For non-ICS patients with ≤ 1 exacerbation in the previous year, patients in the > 150–≤ 300 cells/μL and > 300 cells/μL subgroups had exacerbation RRs of 1.08 (95% CI 0.97, 1.20) and 1.14 (95% CI 1.00, 1.29), respectively, when compared with the ≤ 150 cells/μL group; patients with ≥ 2 exacerbations in the previous year in the > 150–300 cells/μL and > 300 cells/μL subgroups had exacerbation RRs of 1.04 (95% CI 0.88, 1.23) and 0.97 (95% CI 0.79, 1.19), respectively.

**Table 4:** Rate ratio of annual exacerbations combining ICS use and exacerbation history by eosinophil count (cells/μL)

		≤ 150 cells/μL	> 150–≤ 300 cells/μL	> 300 cells/μL
Exacerbation history and ICS use	0 exacerbations and no ICS *n* = 3784	Rate: 0.31	Rate: 0.31 RR: 1.03 (95% CI 0.87, 1.21)	Rate: 0.36 RR: 1.17 (95% CI 0.97, 1.40)
	0 exacerbations and ICS *n* = 3008	Rate: 0.49	Rate: 0.49 RR: 1.00 (95% CI 0.86, 1.16)	Rate: 0.55 RR: 1.12 (95% CI 0.94, 1.33)
	1 exacerbation and no ICS *n* = 3378	Rate: 0.48	Rate: 0.55 RR: 1.15 (95% CI 1.00, 1.32)	Rate: 0.52 RR: 1.09 (95% CI 0.91, 1.29)
	1 exacerbation and ICS *n* = 6381	Rate: 0.69	Rate: 0.72 RR: 1.04 (95% CI 0.95, 1.13)	Rate: 0.76 RR: 1.10 (95% CI 1.00, 1.22)
	≤ 1 exacerbation and no ICS *n* = 7162	Rate: 0.39	Rate: 0.42 RR: 1.08 (95% CI 0.97, 1.20)	Rate: 0.44 RR: 1.14 (95% CI 1.00, 1.29)
	≤ 1 exacerbation and ICS *n* = 9389	Rate: 0.62	Rate: 0.64 RR: 1.02 (95% CI 0.95, 1.10)	Rate: 0.68 RR: 1.10 (95% CI 1.00, 1.19)
	≥ 2 exacerbations and no ICS *n* = 1668	Rate: 0.79	Rate: 0.82 RR: 1.04 (95% CI 0.88, 1.23)	Rate: 0.76 RR: 0.97 (95% CI 0.79, 1.19)
	≥ 2 exacerbations and ICS *n* = 3906	Rate: 1.13	Rate: 1.24 RR: 1.09 (95% CI 1.00, 1.19)	Rate: 1.21 RR: 1.07 (95% CI 0.96, 1.19)

RR versus ≤ 150 cells/μL eosinophil subgroup.

*CI* Confidence interval; *ICS* Inhaled corticosteroids; *RR* Rate ratio.

In patients treated with ICS at baseline, we identified higher annual exacerbation rates for each of these analyses compared with their non-ICS equivalents (Table 4). For ICS patients with ≤ 1 exacerbation in the previous year, patients in the > 150–≤ 300 cells/μL and > 300 cells/μL subgroups had exacerbation RRs of 1.02 (95% CI 0.95, 1.10) and 1.10 (95% CI 1.00, 1.19), respectively, when compared with the ≤ 150 cells/μL group; patients with ≥ 2 exacerbations in the previous year in the > 150–300 cells/μL and > 300 cells/μL subgroups had exacerbation RRs of 1.09 (95% CI 1.00, 1.19) and 1.07 (95% CI 0.96, 1.19), respectively.

## Discussion

In this analysis, which focuses on the value of blood eosinophil counts as a biomarker for future exacerbations, we did not find a strong association between blood eosinophil counts and annual exacerbation rates during the study period; this was true regardless of exacerbation history or ICS use. There was a pattern across the majority of the subgroups analysed, demonstrating that the lowest exacerbation rates were observed in patients with ≤ 150 cells/μL. However, the increases in exacerbation rate observed with increasing eosinophil count were small, with RRs between 0.97 and 1.17.

In line with previous studies, ICS users experienced a higher rate of exacerbations compared with non-ICS users, most likely due to bias by indication, because these patients were receiving ICS as a result of previous exacerbation events [12]. Similarly, patients with a history of ≥ 2 exacerbations in the previous year also experienced a higher rate of exacerbations (range 1.02–1.10) during the study period than patients with ≤ 1 exacerbation in the previous year (range 0.51–0.57). This is consistent with previous studies reporting that the best predictor of future exacerbation risk is the patient’s exacerbation history [23]. Overall, using exacerbation history or ICS use as stratification factors resulted in an increase in the observed exacerbation rate. We observed over 100% increase in exacerbation rate in patients with ≥ 2 exacerbations in the previous year compared with patients with no exacerbations in the previous year. In contrast, only around a 10% increase was noted when comparing the lowest eosinophil count with the higher eosinophil counts.

Several analyses of data from RCTs have investigated the relationship between blood eosinophils and ICS use, and have shown that blood eosinophil count correlates with clinical response to ICS with regards to reducing exacerbation rates [20, 33–35]. These study designs include ICS withdrawal for many patients, where treatment differences become apparent at higher blood eosinophil counts; an association between exacerbation rates and blood eosinophil counts can then be more clearly observed in patients not receiving ICS. In the current analysis, none of the 11 individual studies, and by extension the pooled analysis, were designed to study the relationship between blood eosinophils as a predictor of response to ICS in patients experiencing exacerbations, and therefore this topic is beyond the scope of the current manuscript. In the pooled population, which included patients with stable ICS use or non-use, we did not find a strong association between blood eosinophil counts and annual exacerbation rates.

Some cohort studies have also shown an association between blood eosinophil counts and exacerbation risk. Vedel-Krogh et al. found that individuals with a blood eosinophil count above 340 cells/μL had a higher risk of severe exacerbations compared with patients below this value (odds ratio 1.76; 95% CI 1.56, 1.99) [15]. In addition, Yun et al. found that patients with blood eosinophil counts ≥ 300 cells/μL had an increased risk of exacerbations in the COPDGene study [17]. However, a retrospective study of 992 patients with COPD by Adir et al. found that, among patients who experienced severe exacerbations (72%) and those that did not (71%), there was no difference in the proportion of patients with eosinophil counts of ≥ 2% (*P* = 0.93) [18]. Similarly, a recent population-based study in 57,209 patients with COPD did not find any relationship between blood eosinophil count and risk of exacerbations [19].

As eosinophil levels are known to vary over time, this may lead to patients fluctuating between different eosinophil categories, despite the patient experiencing only a small absolute change [36, 37]. This may, at least in part, explain why the relationship is difficult to conclude.

When assessing eosinophils in categories, the current study did not show a strong relationship between blood eosinophil counts and risk of future exacerbations. Although there is a paucity of data evaluating blood eosinophil count as a continuous value, Bafadhel et al. [20] modelled eosinophil count as a continuous variable and showed an increase in exacerbation rate in line with increasing eosinophil count in patients not receiving ICS. In clinical practice, eosinophils alone should not be used as a predictor of exacerbation risk, but as a potential biomarker of response to ICS in patients with increased exacerbation risk [20, 33–35].

Other candidate biomarkers have been studied as predictors of increased risk of COPD exacerbations [3, 9–11], including bone morphogenic protein-3, Cerberus 1, inhibin B, thrombopoietin, matrix metalloproteinase-10 and a number of interleukin family members [11]. In patients with stable COPD, elevated levels of fibrinogen have been associated with an increased risk of frequent exacerbations [9, 38].

In RCTs, blood eosinophil counts > 300 cells/μL are associated with the greatest effect of ICS on exacerbation prevention. In this analysis, 20.1% had eosinophil counts above this threshold. These results are in line with other studies that suggest that around 25% of patients have eosinophil counts higher than 300 cells/μL [12, 19, 39].

Our analysis has some advantages. Since the pooled studies were all conducted by one pharmaceutical company (Boehringer Ingelheim), the methodologies between these studies are generally compatible. Due to the pooled population, this analysis has a very large sample size and is sufficiently powered to detect even modest changes in exacerbation frequency. However, similar to observational studies, post hoc analyses should be treated with caution as these may be subject to bias. Secondly, the inclusion and exclusion criteria between the studies are mostly generalisable; however, additional factors such as comorbidities may be present in some of the pooled patient population, which were not controlled for and may introduce variability. Thirdly, the pooled studies included a number of active-controlled, or placebo-controlled studies. In COPD, active-controlled studies compare the drug of interest against a leading comparator in the field. Although more ethical, the results of the active-controlled trials could impact on the overall exacerbation rates. It is important to note that this pooled analysis was not designed to assess a relationship between blood eosinophil count and response to ICS use. The scope of this study was to determine the use of eosinophils as a predictor of exacerbations.

What are the practical implications of our study? For many of the currently studied biomarkers of exacerbation risk, the data are conflicting [40]. Whilst blood eosinophils are recommended as a predictor of likely response to ICS, the results from this pooled analysis indicate that eosinophil levels cannot be confidently used as a predictive marker for rate of future COPD exacerbations, as we were unable to identify a strong relationship between the rate of exacerbations and eosinophil levels. In the absence of a clear biomarker to predict the risk of future exacerbations among patients with COPD, previous history of exacerbations continues to be the strongest predictor of future exacerbation risk [17].

## Conclusions

In this pooled analysis of 22,125 patients with COPD, we did not find a clinically important relationship between baseline blood eosinophil count and exacerbation rate. This analysis, coupled with other studies on this topic [18, 19], indicate that blood eosinophil counts are not a clinically useful predictor of future exacerbation risk.

## Data Availability

The datasets generated during and/or analysed during the current study are available from the corresponding author on reasonable request.

## Abbreviations

CI: Confidence interval
COPD: Chronic obstructive pulmonary disease
FEV_1_: Forced expiratory volume in 1 s
GOLD: Global Initiative for Chronic Obstructive Lung Disease
ICS: Inhaled corticosteroids
RCT: Randomised controlled trial
RR: Rate ratio
